# Evolutionary and developmental considerations of the diet and gut morphology in ceratophryid tadpoles (Anura)

**DOI:** 10.1186/s12861-020-00221-5

**Published:** 2020-07-29

**Authors:** Marissa Fabrezi, Julio César Cruz

**Affiliations:** Instituto de Bio y Geociencias del NOA, CCT CONICET Salta-Jujuy, Rosario de Lerma, Salta, República Argentina

**Keywords:** Plasticity, Size, Metamorphosis, Larvae, Horned frog, Carnivory

## Abstract

**Background:**

Before metamorphosis, almost all anuran tadpoles are omnivores. Larval carnivory occurs in some species and, it is associated with distinctive morphotypes. Obligatory carnivorous tadpoles exhibit structural changes in the gastrointestinal tract compared to larvae that are predominately omnivores. The most distinctive feature of the anuran family Ceratophyridae (three genera) overall is the enormous gape of adults. This feature increases their ability to capture extremely large and active prey. The larvae of Ceratophyrid genera are remarkably distinct from each other and carnivory has diversified in a manner unseen in other anurans. The larvae of one genus, *Lepidobatrachus*, has a massive gape like the adult. Herein, we report on larval developmental variation, diet, gross morphology of the gastrointestinal tract, and histology of the cranial segment of the gut before, during and after metamorphosis in larval series for the following ceratophryid species: *Chacophrys pierottii, Ceratophrys cranwelli, Lepidobatrachus laevis* and *Lepidobatrachus llanensis*.

**Results:**

We described patterns of larval development with variation in growth with consequence to the final size at the end of metamorphosis. These patterns seem to be influenced by food quantity/quality, and most predominant by animal protein. Prey items found in pre and post-metamorphic *Lepidobatrachus* spp. are similar. Tadpoles of *Ceratophrys* and *Chacophrys* (and other anurans) share a short cranial segment of the gut with an internal glandular, mucous secreting epithelium, a double coiled intestine and the sequence of metamorphic changes (tract is empty, the stomach differentiates and the intestine shortens abruptly). In contrast, *Lepidobatrachus* tadpoles have a true stomach that acquires thickness and increased glandular complexity through development. As larvae they have a short intestine without double coils, and the absence of intestine shortening during metamorphosis.

**Conclusions:**

The larval development of the gastrointestinal tract of *Lepidobatrachus* is unique compared with that of other free-living anuran larvae. An abrupt metamorphic transformation is missing and most of the adult structural features start to differentiate gradually at the beginning of larval stages.

## Background

Metamorphosis is a period of irreversible, dramatic ontogenetic change from a multicellular, free-living, post-embryonic stage (“larva” in animals) to a multicellular, pre-reproductive adult (“juvenile” in animals); it is also associated with a change in habitat [1]. Before metamorphosis, almost all anuran larvae are omnivores. After metamorphosis, all anurans are carnivorous. Metamorphosis of the digestive system involves a transformation that includes the disappearance of the anal tube, extensive shortening of the alimentary tract, and differentiation of a stomach with the glandular complexity necessary to process a diet based on animal protein.

Larval carnivory occurs in some species and, where it is common, it is associated with distinctive morphotypes [2]. Obligatory carnivorous tadpoles have a gastrointestinal tract that differs in structure from that of larvae that are predominately omnivores. Furthermore, a diet based on animal protein enhances development in carnivorous tadpoles [3, 4].

Frogs of the South American family Ceratophryidae (three genera: *Chacophrys*, *Ceratophrys*, *Lepidobatrachu*s) are distinguished by robust bodies, short limbs, and massive heads, with an exaggerated mouth in which a pair of fangs protrude from the lower jaw. These features are associated with an evolutionary shift in the Ceratophryidae toward feeding on large prey, which includes many arthropod taxa, mollusks, anurans, snakes, lizards, birds and small mammals [5].

Tadpoles of ceratophryid genera are remarkably distinct, but they share accelerated developmental rates compared to tadpoles that are omnivorous and other taxa with obligated carnivorous tadpoles [6–9]. Carnivory has diversified in a manner unseen in other anurans since the *Lepidobatrachus* tadpole (with a massive gape) has become more like an adult [10].

*Chacophrys* has an omnivorous tadpole that feed on plankton and detritus. Its larval development occurs in just 2 weeks, with snout-vent length at the end of metamorphosis ranging from 20 to 45 mm (this article, [11]). Tadpoles of *Ceratophrys* are highly specialized carnivores with robust and sharp keratinized jaws. Larval development in *Ceratophrys cranwelli* takes 3 weeks, and snout-vent length varies between 30 and 40 mm at metamorphosis [12]. *Lepidobatrachu*s larvae are suctorial with a huge oral cavity. They swallow very large, living nekton including other tadpoles [6]. After 2 weeks as larvae, newly metamorphosed *Lepidobatrachus* reaches snout-vent length between 40 and 50 mm [12, 13]. As previous authors have suggested, *Lepidobatrachus* has a life style in which larval and adult stages share a unique ecomorphology [9, 10]. *Lepidobatrachus* larvae have a short alimentary tract with a dilated cranial segment with a distinct glandular surface that is both structurally and functionally like the adult stomach. This includes a pepsinogen in early development that is electrophoretically indistinguishable from a form found in postmetamorphic *Lepidobatrachus* [14]. Evolutionary novelties in *Lepidobatrachus* larvae include a capacious stomach, the greater ratio of foregut to hindgut than any omnivorous tadpoles, and a gastroduodenal loop in a more caudal position [15, 16].

Much is known about the morphology and ecology of the ceratophryids, in both larval and adult stages, but there is little comparative information on how modification of a common ancestral developmental pathway played a role in shaping their particular ontogenies. Here, from field specimens and field observations we provide data on 1) larval variation, 2) diet, 3) gross morphology, and 4) microscopic features of the anterior gastrointestinal before, during and after metamorphosis. Our results on ontogenetic variation in the gastrointestinal tract among ceratophryids provide insights to the evolution of the reduced ecomorphological differences between larvae and adults in *Lepidobatrachus.*

## Results

### Intraspecific and interspecific developmental variation

Larval development for *Chacophrys pierottii* and *Ceratophrys cranwelli* (as representative species in the two genera) follows the sequence in the Gosner table [17]. Stages 26–36 correspond to the proximo-distal differentiation of the hind limb followed by the postaxial-preaxial differentiation of autopodium. Stages 37–40 follow the appearance of tubercles in digits and limb growth. At Stage 41 the vent tube is lost. Stage 42 is diagnosed by forelimb emergence, loss of the larval denticles and horny beaks, and the cessation of larval growing. As most anurans, metamorphosis starts at this stage. Stages 43–46 are characterized by changes in the chondrocranium that convert the small-mouthed tadpole to a large-mouthed frog. During these stages, the tail shortens and finally disappears.

*Lepidobatrachus* spp. larvae lack digital tubercles and horny beaks. Their forelimbs develop externally. These features are so unusual that it is difficult to align *Lepidobatrachus* stages of limb growth and the beginning of the metamorphosis with standard Gosner stages. The major feature indicating metamorphosis in *Lepidobatrachus* is the degree of vent tube shortening and the formation of a cloaca. Other features that indicate the start of metamorphosis in the genus are the fusion of spiracle flaps with the forelimb skin and the disappearance of keratinized larval teeth.

**Fig. 1:**
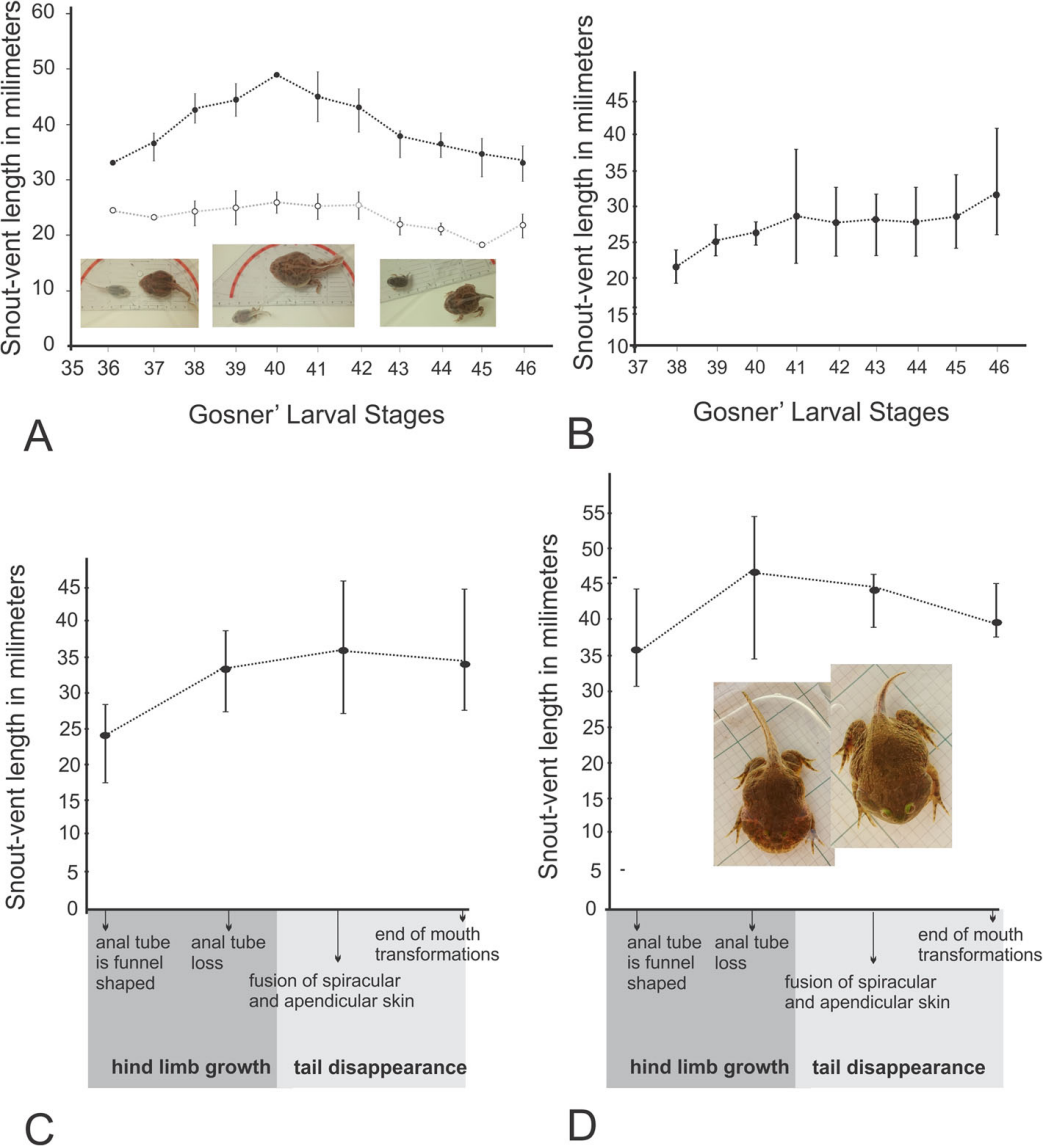
Larval growth in ceratophryid species. **a**. *Chacophrys pierottii* has two growth trajectories. Large tadpoles metamorphose in two weeks. This size variation could be influenced by food quality and/or pond desiccation. **b**. *Ceratophrys cranwelli* also has accelerated growth compared to non-ceratophryids species with a rapid larval development completed in three weeks. **c**. *Lepidobatrachus llanensis* and **d**. *L. laevis. Lepidobatrachus* spp. have particularly large and fast-growing tadpoles. Tadpoles of *Lepidobatrachus* reach the end of metamorphosis in two weeks. Intraspecific size variation seems to be related to food quantity. Ceratophryid frogs all reproduce explosively once a year at the beginning of the wet and warm season

Accelerated developmental rates in ceratophryid tadpoles described in previous studies (summarized in [9] and growth trajectories indicated they reach the metamorphosis at the minimum size of 30 mm of snout-vent (Fig. 1). However, there is a developmental series of *Chacophrys pierottii* with tadpoles metamorphosing at a smaller size (Fig. 1a). Small and large tadpoles belong to different clutches revealing an amplitude in size variation is greater to than reported previously [9].

### Tadpole diets and gut contents through metamorphosis

**Fig. 2:**
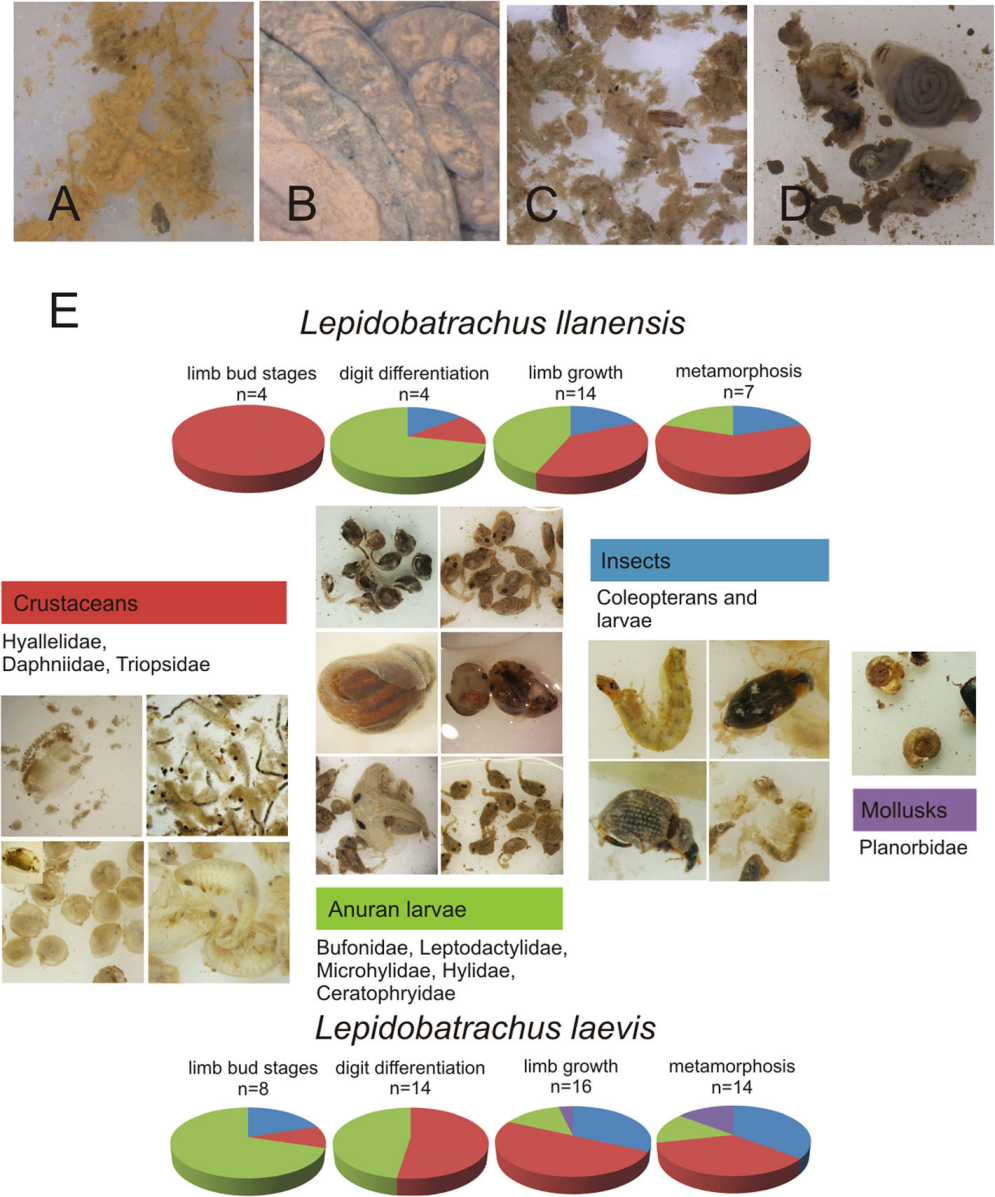
Gut contents in ceratophryid tadpoles. **a**. Food content in the foregut of the small tadpole (Stage 37) of *Chacophrys pierottii*, the presence of small particles of detritus and macerated shrimp are observed. **b**. Ventral view of the coiled gut in a large tadpole of *Ch. pierottii* (Stage 37)*.* Shrimps are observed by transparency. **c**. Detail of the macerated shrimp, pieces of wood and filamentous of algae in the gut of the specimen shown in **b**. **d**. Gut content in a tadpole of *C. cranwelli* (Stage 37), in which pieces of tadpoles are predominant. **e**. Diet during larval stages in *Lepidobatrachus llanensis* and *L. laevis.* Stomachs are full of food at all larval stages in both species. Furthermore, the whole alimentary tracts contain digested organic matter. Crustaceans and tadpoles are frequent. Occasionally, tadpoles of these species may eat insects and snails

Small tadpoles of *Chacophrys pierottii* graze on the pond bottom and their gut contents reveal principally detritus with pieces of dark organic fragments such as wood, shrimp eyes, insect pieces (Fig. 2a). Whereas large tadpoles feed actively in the water column and their gut contents show remnants of shrimp and algae (Fig. 2b, c). These tadpoles stop feeding when the forelimbs emerge and they shed their keratinized horny beaks (Stage 42). Gut contents diminish in a caudal direction and at Stage 44 the whole gastrointestinal tube is empty. When metamorphosis is complete, froglets (from large tadpole series) have empty guts (*n* = 5) whereas one contained eight *Ch. pierottii* tadpoles, and others (*n* = 2) insects (larvae and adult coleopterans). The cannibalism suggests emerging froglets forage at the margins of the pond.

Before metamorphosis, tadpoles of *Ceratophrys cranwelli* have an anterior stomach-like dilatation full of pieces of tadpoles (Fig. 2d), shrimp, and insects with the intestine filled with disassembled remnants of food. Tadpoles of *C. cranwelli* also stop feeding when their forelimbs emerge and they shed their keratinized beaks. Gut contents are reduced at Stage 43 and completely absent by Stage 44.

In contrast, for *Lepidobatrachus laevis* and *L. llanensis* tadpoles, ranging from larval limb bud stages to when their tails completely regressed (Stage 27–46) we always found prey items in their stomachs, which suggests that they do not stop feeding through metamorphosis. Prey items for both species included crustaceans, anurans, and insects (Table 1). *L. laevis* larval gut contents also included some snails.

**Table 1:** Number of prey during different life larval stages in *Lepidobatrachus* spp.

Species	Stages	n	Anurans	Crustaceans	Mollusks	Insects
*Lepidobtrachus laevis*	Limb bud	8	*Rhinella major* (74), *Leptodactylus latinasus* (26), *Lepidobatrachus laevis* (1)	*Daphnia* (1)	–	Larvae (2)
	Digit differentiation	7	*Rhinela schneideri* (2), *R. major* (15), *Leptodactylus bufonius* (3), *Scinax* sp. (5), *Dermatonotus muelleri* (10), *Pseudis paradoxa* (3), *Lepidobatrachus laevis* (1)	*Daphnia* (13), *Hyallela* (15), *Triops* (1)	–	–
	Limb growth	19	*Scinax* sp. (8), *Leptodactlus bufonius* (3)¸ *Dermatonotus muelleri* (3), *Rhinella major* (2), *R. schneideri* (1), *Pseudis paradoxa* (2), *Physalaemus biligonigerus* (1) *Ceratophrys cranwelli* (1)	*Daphnia* (25), *Hyallela* (35), *Triops* (3)	*Biomphalaria* (2)	Hemiptera (2), Coleoptera (5), Hymenoptera (1)
	Metamorphosis	14	*Scinax* sp. (4), *Dermatonotus muelleri* (2), *Physalaemus biligonigerus* (6)	*Hyallela* (10), *Daphnia* (18), *Triops* (1)	*Biomphalaria* (3)	Coleoptera (16) Larvae (2)
*Lepidobtrachus llanenesis*	Limb bud	4	–	*Hyallela* (17)	–	–
	Digit differentiation	4	*Chacophrys pierottii* (6), *Leptodactylus* sp. (24)	*Hyallela* (29), *Daphnia* (7)	–	Larvae (2)
	Limb growth	14	*Chacophrys pierottii* (2), *Leptodactylus bufonius* (3), Indet (2)	*Hyallela* (39), *Daphnia* (44), *Triops* (1)	–	Coleoptera (3), larvae (3)
	Metamorphosis	7	*Chacophrys pierottii* (1)	*Daphnia* (12)	–	Coleoptera (5)
			Indet (2)	*Hyallela* (70)		Larvae (1)

The crustaceans *Daphnia* sp. and *Hylallela* sp. and anuran tadpoles were the dominant stomach contents in *Lepidobatrachus* larvae. These prey were predominant in the diet at all larval stages (Fig. 2e). At a similar stage and size, stomachs contents may present few and large prey (tadpoles) or much small prey (*Hyallela* sp.) suggesting there is no correlation between the size or stage of the tadpoles and the size of the prey that they had in their stomachs. The largest prey consumed are the crustacean *Triops* and tadpoles of various species (Table 1).

The stomach contents provide some indication of where *Lepidobatrachus* tadpoles feed. Tadpoles of *Physalaemus biligonigerus, Rhinella major*, and a metamorphosing *Chacophrys pierottii* (in the stomach of an advanced tadpole of *L. llanensis*) were likely captured near the edge of the pond where the water is shallow. Other prey are nektonic, consistent with the *Lepidobatrachus* tadpoles hunt in the water column.

We identified one case of cannibalism where a *L. laevis* eat a *L. laevis* tadpole and one case in which a *L. laevis* preyed upon on a *Ceratophrys cranwelli* tadpole. *Ch. pierottii* were found in the stomachs of *L. llanensis*. Small juvenile snails (*Biomphalaria* sp.) were present in the gut of *L. laevis* larvae at later stages.

### Visceral gross morphology

Ceratophryid tadpoles share the following features of visceral gross morphology: 1) lungs are well developed and inflated early, at limb bud stages; 2) fat bodies are absent during larval development; and 3) gonads remain undifferentiated up to froglet stages.

**Fig. 3:**
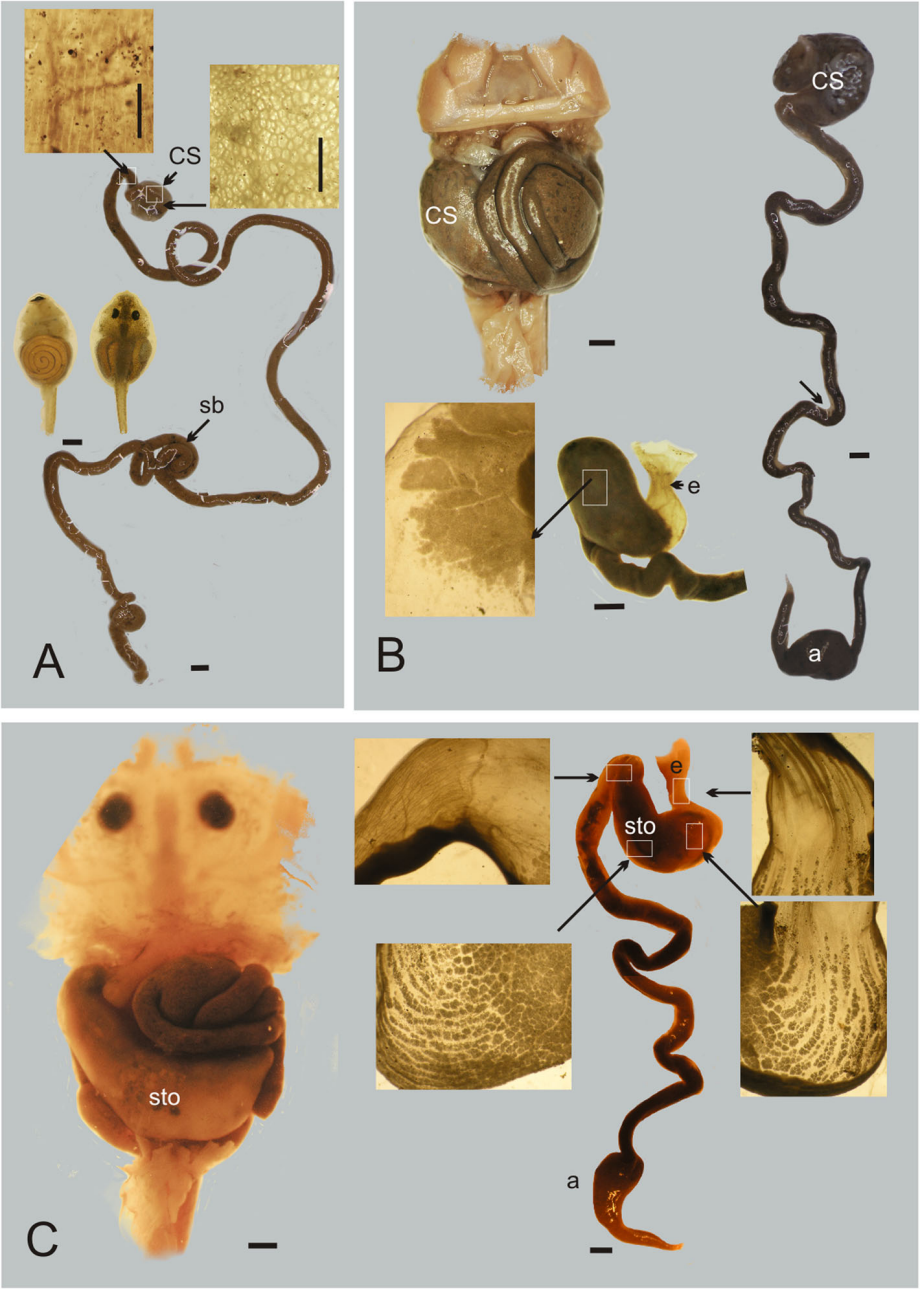
Gross morphology of the gastrointestinal tract within ceratophryids. **a**. Tadpole of *Chacophrys pierottii* at Gosner Stage 29, ventral and dorsal views showing the abdominal cavity and the coiled gastrointestinal tube. The long intestine shows little regional specialization, with a short anterior segment that has a reticulated surface different from the intestine that is smooth with longitudinal stretches. **b**. Tadpole of *Ceratophrys cranwelli* at Gosner Stage 30*,* visceral morphology in ventral view. The gastrointestinal tract is shorter with differentiated anterior sac and the ampulla of the rectum. The esophagus has longitudinal folds and it is dorsal in respect to the stomach. The adult-like stomach is transparent with a “glandular” surface. **c**. Tadpole of *Lepidobtrachus llanensis* at Gosner Stage 32*,* the stomach occupies the half of the abdominal cavity. The gastrointestinal tract is short. The esophagus is dorsal and has longitudinal folds. The stomach has a dense pattern of glands and the gastrointestinal loop delimits the stomach from the pyloric region. The epithelium of the intestine is smooth with some aligned cells

In *Chacophrys pierottii* tadpoles (Stages 27–41), the coiled gut occupies almost the whole pleuroperitoneal cavity. It is visible through the skin in ventral view (Fig. 3a). In dorsal view, the liver and the pancreas are observed on the right side embraced by the gastroduodenal loop. This loop, which is in a relatively anterior position, is the most cephalic curvature of the gut: it delimits the esophagus and the cranial segment of the gut considered a larval “stomach” (i.e. the *maniccotto glandulare,* with a distinct internal glandular surface) from the intestine. The intestine is very long (Table 2), double spiralled, and arranged in a three-dimensional configuration of loops and coils. The intestine is uniform in diameter, its epithelium is corrugated and it is filled with organic matter.

**Table 2:** Variation of body length and intestine length (in millimeters) during larval development within Ceratophryids

Species	Stage	n	Body Length (BL)	Hind Gut Length (HGL)	HGL/BL
*Chacophrys pierottii*	Limb bud	5	9,22 (±0.53)	95.64 (±15.61)	≈ 10
	Limb growth	5^a^	25.43 (±1.84)	305.58 (±92.5)	≈ 12
		5^b^	38.25 (±4.28)	633.6 (±196.72)	≈ 16.56
	Metamorphosis	3^a^	23.84 (±3.20)	172.88 (±67.47)	≈ 7.4
		3^b^	36.51 (±1.73)	120.06 (±75.62)	≈ 3.2
*Ceratophrys cranwelli*	Limb bud	6	11.73 (±1.40)	51.39 (±10.05)	≈ 4.5
	Limb growth	6	23.80 (±1.45)	81.94 (±8.4)	≈ 3.5
	Metamorphosis	2	28.77	52.5	≈ 1.8
*Lepidobatrachus llanensis*	Limb bud	5	13.36 (±1.12)	33.89 (±3.34)	≈2
	Limb growth	10	25.062 (±5.17)	51.83 (±14.80)	≈2
	Metamorphosis	10	40.26 (±4.8)	118.55 (±22.86)	≈ 2.9
*Lepidobatracus laevis*	Limb bud	6	13.85 (±2.61)	27.05 (± 4.80)	≈ 2
	Limb growth	15	37.38 (±6.11)	102.59 (± 30.64)	≈ 2.7
	Metamorphosis	17	46.17 (±4.06)	131.65 (±17.30)	≈ 2.85

In *Chacophrys pierotti,* super index (^a^) refers to small tadpoles and super index (^b^) to large tadpoles. Length of intestine was measure between the pyloric loop and end of the intestine into the vent tube/cloaca. Before the metamorphosis, the intestine is very large in *Chacophrys pierottii*, short in *Ceratophrys cranwelli* and very short in *Lepidobatrachus* spp. During metamorphosis, the intestine shortens abruptly in *Chacophrys* and *Ceratophrys* whereas it conserves its larval length in *Lepidobatrachus* spp

Beginning with forelimb emergence, the gut proceeds to differentiate as follows: 1) the intestine is empty; 2) the diameter of the intestine diminishes posteriorly (from stage 42), 3) there is an abrupt shortening of the intestine (from stage 43) (Table 2); 4) the stomach differentiates (from stage 44), and 4) the rectum (different in diameter from the majority of the intestine) expands to form the ampulla.

The gastrointestinal tract in tadpoles of *Ceratophrys cranwelli* (Stages 26–41) presents a dilated stomach-like region, and a well-formed ampulla in the posterior intestine (Fig. 3b). The esophagus is a tube with longitudinal folds indicating a capacity to ingest a bolus of large diameter. The dilated foregut lies longitudinally, its wall is transparent with a glandular internal surface. The intestine is shorter than in *Ch. pierottii* (Table 2), double spiralled, and arranges in a three dimensional configuration of loops and coils. The ampulla of the hindgut is hemispheric and it is visible at left. Beginning with forelimb emergence the sequence of changes is as follows: 1) the intestine is emptying; 2) the diameter of the intestine diminishes; 3) the stomach differentiates, and 4) the intestine shortens (Table 2).

In *Lepidobatrachus laevis* and *L. llanensis* the stomach and the ampulla of the rectum occupy half of the pleuroperitoneal cavity as seen in the ventral view (Fig. 3c). The esophagus is a tube with longitudinal folds indicating it can expand to accommodate a large bolus of ingested material. The stomach is a dilated, long, transversally sac oriented with an internal glandular fundic area. The walls of the stomach increase thickness near the metamorphosis. The stomach continues as the duodenum separated by pyloric constriction. The intestine is shorter than in *C. cranwelli* (Table 2)*,* it is arranged in a three-dimensional configuration of disorganized loops. The diameter of the intestine is uniform with irregular contents of digested organic matter. The ampulla of the rectum is oblong and it is visible anteriorly contiguous to the heart. These features are observed from early tadpoles up to postmetamorphic stages without changes in the intestine length (Table 2).

### Microscopic morphology of the anterior segment of the gastrointestinal tract

**Fig. 4:**
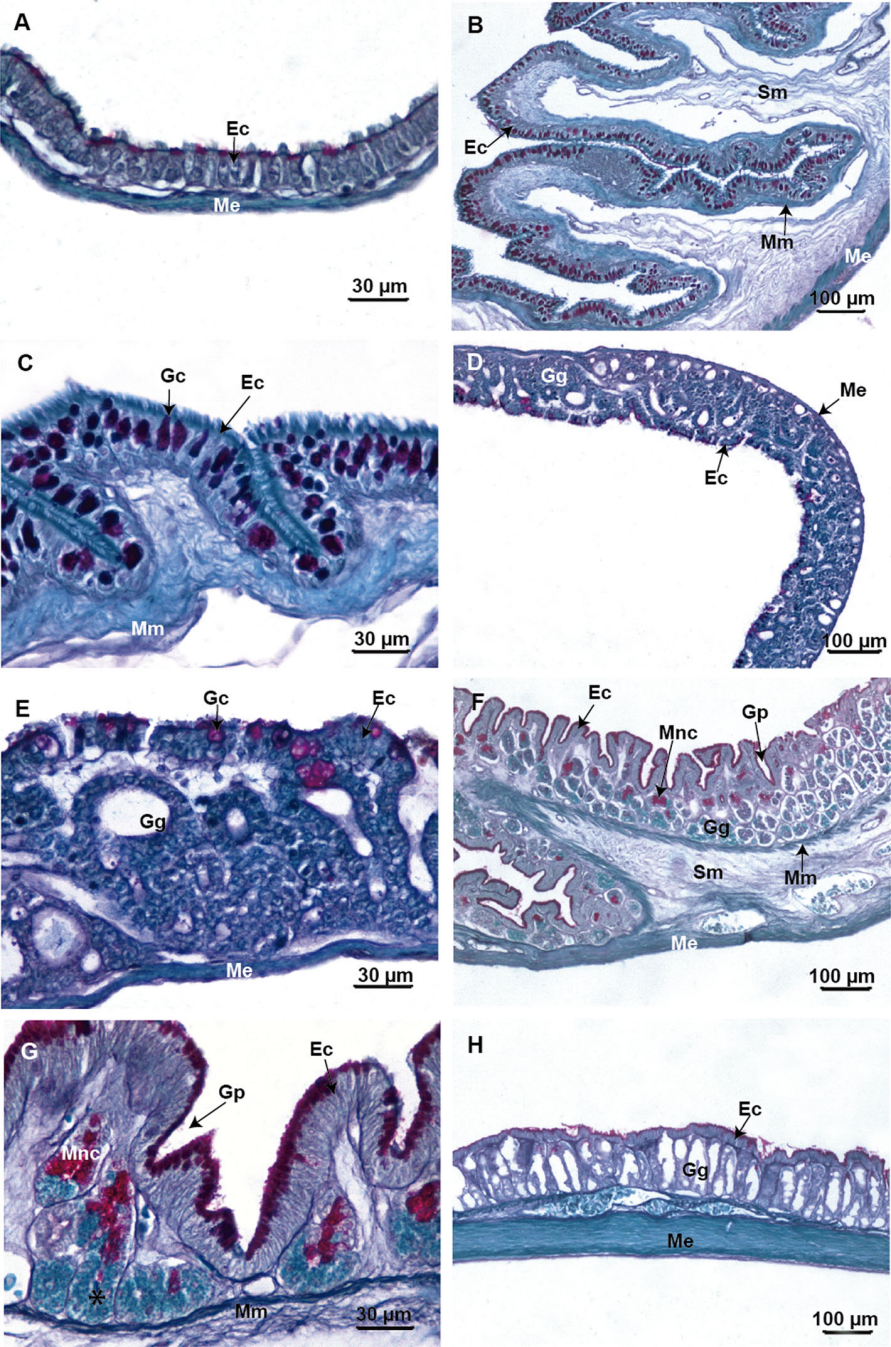
Light microscopy micrographs of the foregut of *Chacophrys pierottii*. **a**. Section of the esophagus in an advanced larval stage (Stage 39) shows a thin mucosa with ciliated cells and columnar mucous cells (PAS-positive). **b** and **c**. Panoramic and detailed sections of the esophagus in a froglet showing folds of mucosa lined by a ciliated epithelium with numerous goblet cells. **d** and **e**. Panoramic and detailed sections of the larval “stomach” (i.e. the *maniccotto glandulare*) exhibit the fundic region with numerous gastric glands and some goblet cells (PAS-positive) in an advanced larval stage (Stage 39). **f**. Middle stomach section with folded mucosa and numerous gastric glands with different types of glandular cells in the froglet. **g**. Amplification of F-image showing gastric glands with mucous neck cells (PAS-positive) at the upper portion and oxynticopeptic cells (*) at the lower portion. **h**. Section at the level of the pyloric region there are small simple tubular gastric glands in the froglet. Abbreviations: Ec: epithelial cells; Gc: goblets cell; Gg: gastric glands; Gp: gastric pits; Me: muscularis externa; Mm: muscularis mucosa; Mnc: mucous neck cells; Sm: submucosa

In *Chacophrys pierottii* the larval esophagus shows a distended and thin mucosa, lined by a ciliated pseudostratified epithelium (Fig. 4a). Mucous secretory columnar cells positive for PAS staining are present in the epithelium indicating the secretion of neutral mucopolysaccharides. The submucosa layer is reduced, and the outer circular muscular layer is evident. In contrast, in postmetamorphic specimens, the epithelium forms a well-folded mucosa (Fig. 4b, c). In addition, small goblet cells concurrently stain positively for both PAS and AB confirming the presence of both acidic and neutral mucopolysaccharides. On the other hand, larger goblet cells are only positive for PAS, indicating the secretion of only neutral mucopolysaccharides (Fig. 4c). There are no tubular glands and the muscularis mucosa is sparse. The submucosa layer is wide and made up of loose connective tissue. External musculature is subdivided into a thick circular layer and a thin longitudinal layer.

The larval anterior stomach (*manicotto glandulare*) shows a fundic region. The mucosa is slightly folded and lined by a columnar epithelium with a brush border (Fig. 4d and e). Few goblet cells are observed; they stain purple with PAS. Tubular glands are observed, but they do not stain with either PAS or AB. The submucosa is not evident, and the outer circular muscular layer is present (Fig. 4e).

In postmetamorphic specimens there is an anterior region that transitions from an esophageal-like region to a fundic region. The middle portion of the stomach has almost entirely the histology of the fundic zone with a folded mucosa (Fig. 4f). The epithelium has secretory columnar cells with a brush border. Apical portion of the epithelial cells is reactive for both PAS and AB stains. There are tubular gastric glands that show mucous neck cells positive for PAS. Zymogen cells are observed mainly in the lower portion of the gastric glands. These cells have granules that stain differently with Masson’s trichrome or with light green (PAS/AB technique background dye) (Fig. 4g). The more caudal region of the stomach has pyloric features (Fig. 4h) with a homogeneous mucosa and shallow folds. The epithelium is made up of mucous secretory columnar cells that stain positively with PAS staining. Tubular glands are observed, but these are negative for combined PAS and AB staining. The layer muscularis externa is made up of a thick circular coat and a very thin longitudinal coat.

**Fig. 5:**
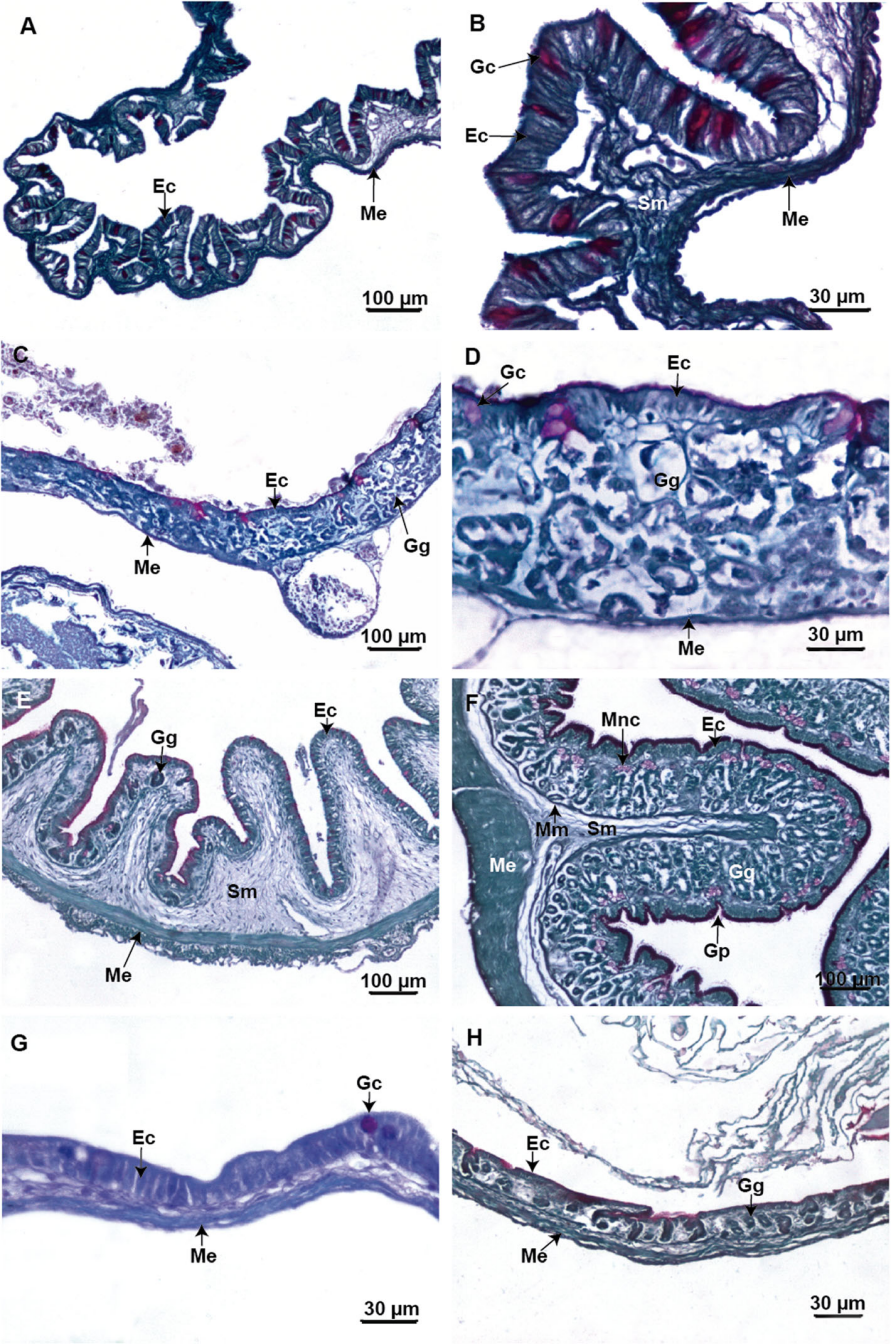
Light microscopy micrographs of the foregut of *Ceratophrys cranwelli*. **a** and **b**. Panoramic and detailed sections of the esophagus in advanced larval stage (Stage 39) showing ciliated and goblets cells (PAS-positive) in the lining epithelium. **c** and **d**. Panoramic and detail sections of the dilated cranial segment of the gut in an advanced larvae stage (Stage 39) to display the fundic region with numerous gastric glands and some goblet cells (PAS-positive). **e**. Sections of the transitional zone between the esophageal-like region and the fundic region with some gastric glands in a specimen at the end of metamorphosis (Stage 46). **f**. Section of the middle stomach showing a folded mucosa and numerous gastric glands in a specimen at the end of metamorphosis (Stage 46). The mucous glands are PAS-positive. **g**. A section at the level of the pyloric region with few PAS-positive goblets cells in advanced larval stage (Stage 39). **h**. Section at the level of the pyloric region has some small simple tubular gastric glands in a specimen at the end of the metamorphosis (Stage 46). Abbreviations: Ec: epithelial cells; Gc: goblets cell; Gg: gastric glands; Gp: gastric pits; Me: muscularis externa; Mm: muscularis mucosa; Mnc: mucous neck cells; Sm: submucosa

In *Ceratophrys cranwelli* the larval esophagus has a mucosa with numerous folds, lined by pseudostratified epithelium with ciliated columnar and goblet cells (Fig. 5a). Goblets cells stain bright purple with PAS. The submucosa layer is thin and is composed of loose connective tissue. The muscularis externa layer involves an inner circular coat and a very thin outer longitudinal coat (Fig. 5b). In contrast, in postmetamorphic specimens the submucosa and the muscularis externa layers are thicker.

The larval anterior stomach shows a fundic region with a distended mucosa (Fig. 5c). The epithelium has mucus secretory columnar cells with a brush border that is slightly PAS and AB positive. Very few PAS positive goblets cells are observed. Tubular gastric glands do not stain differentially with PAS or AB histochemical techniques. The submucosa layer is not evident but there is a thin muscular external layer (Fig. 5d).

In postmetamorphic specimens, the mucosa folds are pronounced and the stomach walls are thickened, including the submucosa and the muscularis externa layers. The cranial portion of the stomach progresses from an esophageal-like surface to a fundic surface similar to the middle stomach (Fig. 5e). The fundic surface has deep gastric pits (Fig. 5f). The epithelial lining is formed by brush-bordered columnar cells whose apical cytoplasm stains violet after combined PAS and AB staining. In addition, the mucosa has simple tubular gastric glands formed by mucous neck cells positive for PAS staining. The lower portion of gastric glands does not differentially stain with PAS/AB, but the cells show cytoplasmic granules with acidophilic characteristics. The muscularis mucosa layer is well defined. The submucosa is made up of loose connective tissue, and the muscularis externa involves an inner thick circular coat and outer longitudinal coat.

The caudal stomach has a distinct pyloric region. In larvae, the mucosa is slightly folded and is lined by a columnar epithelium. Few PAS-positive goblets cells are present (Fig. 5g). In postmetamorphic specimens, the pyloric region shows a slightly folded mucosa lined by an epithelium with mucous secretory columnar cells. In addition, there are small simple tubular gastric glands that do not differentially stain with PAS/AB or Masson’s trichrome (Fig. 5h). Both larvae and post metamorphic specimens have a thin submucosa made up of loose connective tissue. The muscularis externa layer is composed of an inner thick circular layer and an outer thin longitudinal layer (Fig. 5g and h).

**Fig. 6:**
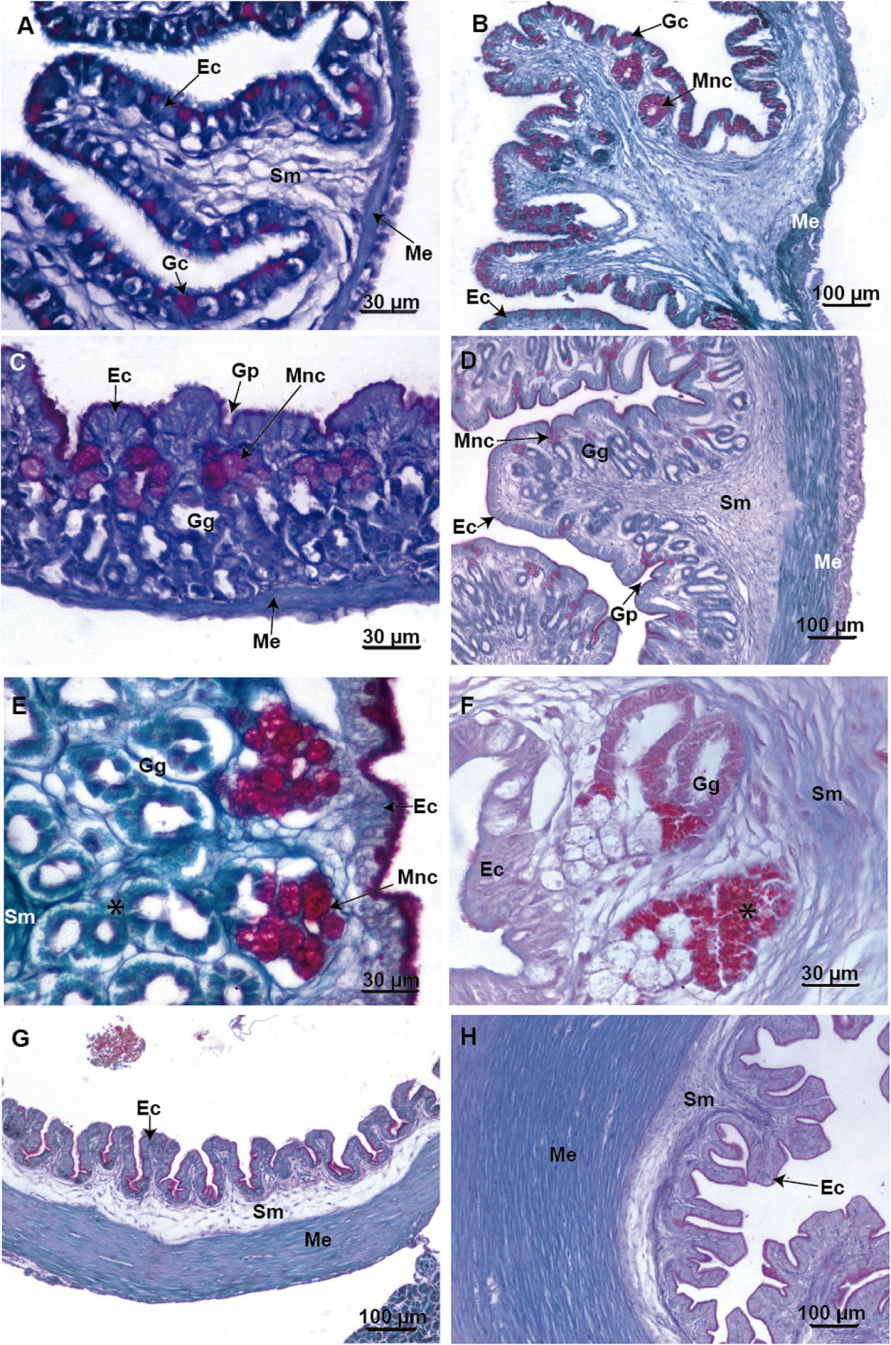
Light microscopy micrographs of the foregut of *Lepidobatrachus* spp. **a**. *L. llanensis.* Section of the esophagus with ciliated cells and numerous goblet cells forming the lining epithelium in an early limb bud larval stage. **b**. *L. llanensis*. Sections of the transitional zone from the esophagus to middle stomach showing gastric glands and the submucosa layer thickened in at the end of larval period. **c**. *L. llanensis*. Section from the middle of the stomach showing different types of glandular cells, and mucous glands already differentiated (PAS-positive) in the fundic region in tadpoles at an early limb bud larval stage. **d**. *L. llanensis*. Section of the middle stomach exhibiting increment of folds, gastric glands, the connective tissue in the submucosa and outer circular muscular layer thickened at the end of larval period. **e**. *L. laevis.* Section of the fundic region displaying simple tubular gastric glands formed by mucous neck cells at the upper portion of the gland and oxynticopeptic cells (*) at the lower portion in an advanced tadpole stage without vent tube. The staining is PAS and AB combined. **f**. *L. llanensis*. Section of the fundic region with granular material of oxynticopeptic cells (*) revealed by Masson’s trichrome method at the end of larval period. **g**. *L. llanensis*. Section at the level of the pyloric region with a remarkable thickness of circular muscles in the muscular layer in an early limb bud larval stage. **h**. *L. llanensis*. Section at the level of pyloric region denotes the increment in the thickness of the wall of the gut at the end of larval period. Abbreviations: Ec: epithelial cells; Gc: goblets cell; Gg: gastric glands; Gp: gastric pits; Me: muscularis externa; mnc: mucous neck cells; Sm: submucosa

In *Lepidobatrachus* spp. the esophagus appears the same in early tadpoles and postmetamorphic specimens. It has a mucosa with numerous folds lined by pseudostratified epithelium with ciliated columnar and goblets cells (Fig. 6a). The number of goblet cells and folds increases during development. The goblets cells appear bright purple after PAS staining. The submucosa layer is composed of loose connective tissue, and no glands are visible in this layer. The muscularis externa layer has circular inner and outer longitudinal fibers. In the cranial region of the stomach an esophageal-like zone and a fundic zone similar to the middle stomach was observed (Fig. 6b).

The middle stomach has a slightly folded mucosa in early tadpoles. At those stages, there is no distinct muscularis mucosa or submucosa (Fig. 6c). Folds are more complex and the gastric pits become deeper as development progresses (Fig. 6d). The mucosa is lined by a secretory columnar epithelium with a brush border. The apical plasma membrane and apical cytoplasm of the epithelial cells appear violet with PAS and AB combined staining indicating the presence of acidic and neutral mucopolysaccharides. In addition, the mucosa layer shows simple tubular gastric glands formed by mucous neck cells that stain bright purple with PAS. Zymogen cells are present mainly in the lower portion of the gastric glands, but they did not stain positively with either PAS or AB (Fig. 6c).

In more advanced developmental stages, the granules in the cytoplasm of zymogen cells stain positive with light green (PAS/AB technique background dye) or Masson’s trichrome, indicating strong acidophilic characteristics (Fig. 6d–f). In addition, a submucosa formed by loose connective tissue is evident. The muscularis externa layer is formed by an outer very thin longitudinal and an inner thick circular coat (Fig. 6d).

The caudal region of the stomach has a distinct pyloric region, with an extensively folded mucosa (Fig. 6g–h). The epithelium is simple and columnar, with mucous secretory cells positive for PAS staining. The submucosa layer is wide and made up of loose connective tissue. The muscularis externa layer is subdivided into a thick circular internal and a very thin longitudinal external layer. The number of folds and the thickness of the inner circular muscle layer increase considerably as the tadpole’s progress toward metamorphosis (Fig. 6h).

## Discussion

### Intraspecific and interspecific developmental variation

Phenotypic plasticity occurs in a stressful environment (e.g. ones with high competition). For example, when tadpoles of species of the spadefoot toads (*Spea bombifrons* and *S. multiplicata*) develop together, they diverge in large carnivorous morphs and smaller omnivorous morphs [2]. For these toads, both field and experimental data provide strong argument supporting the evolutionary consequences of phenotypic and developmental plasticity [18].

Ceratophryid’s development has diversified leading to three different larval phenotypes (based on in trophic morphology and behavior). These are represented by the three extant genera, which may co-occur within the same pond [9]. Differences among the three phenotypes are based on feeding but are also evident in size, some developmental trajectories, and duration of the larval development.

Ceratophryid share rapid growth and the large size at metamorphosis. In the field, larval development is completed in 2 weeks for *Chacophrys pierottii* and *Lepidobatrachus* spp., and 3 weeks for *Ceratophrys cranwelli* [11, 12]. Larval development for most other anurans breeding simultaneously with ceratoprhyid species extends from 20 to 75 days, with smaller sizes at metamorphosis when development is completed in 20 days [13]. Accelerated rates of growth have been reported in the African frog *Pyxicephalus adspersus* that metamorphoses at snout-vent length of 22.4 mm after just 17 days of development [19].

Pond desiccation, competition, food availability, and food quality are factors that can influence the length of the larval period and size at the metamorphosis. Growth trajectories for *C. cranwelli* and *Lepidobatrachus* spp. reveal intraspecific variation that is likely influenced by food quantity. Although we do not have data on developmental time to say whether the smaller tadpoles of *Ch. pierottii* develop faster; they would seem to be predominant detritivorous and benthic. This fact suggests further studies to test experimentally the hypothesis if variation of growth in *Ch. pierottii* would be related to locomotion in drying ponds, and/or food.

Metamorphosis occurs only when the limbs have grown in size and differentiated enough to make terrestrial locomotion possible. In tadpoles of *Ch. pierottii* and *C. cranwelli,* like most anurans, metamorphosis begins the moment the vent tube is lost and the forelimb emerge. In *Lepidobatrachus,* the early loss of the vent tube indicates that remaining metamorphoric changes may promptly follow. This is consistent with that vent tube disappearing as the first sign of metamorphosis when it is experimentally induced with exogenous thyroxine [20].

Then, the accelerated rates of growth among ceratophryid lineages resulted in large size at the metamorphosis and modified developmental trajectories (e.g., earlier anal tube disappearance) that permit the threshold for metamorphose to be reached in exceptional short time.

### Tadpole diets and gut contents through metamorphosis

Tadpole’s diets vary in the relative amounts of protein, carbohydrate, and lipids. Tadpoles of some species may adjust their foraging behavior to better meet their nutritional needs. Nutritional variation, in turn, may influence the size at or time to metamorphosis [21]. Apparently, among ceratophryids diet influences the size at the metamorphosis rather than time.

As was reported previously and noted above, tadpoles of *Ceratophrys* and *Lepidobatrachus* are predators on other anuran larvae and for *Ceratophrys* their diet is predominantly based on tadpoles [5]. Crustaceans however are an important part of *Lepidobatrachus* spp. diet. Stomachs of *Lepidobatrachus* tadpoles may contain a few large prey items or many small prey items, and their stomach contents indicate they are opportunistic predators, often preying on a wide variety of invertebrate (crustaceans, insects, and mollusks), and anuran larvae. Cannibalism is frequent. The absence of fish among their gut contents —even when numerous species of fish co-exist [22] with ceratophryid tadpoles— would indicate they are not able to catch fish; e.g., their speed or mobility protects them *Lepidobatrachus* tadpole predation.

In spite of the voracious feeding behavior, megalophagy and cannibalism attributed to adult Ceratophryidae, they are most likely generalist, and opportunistic predators, capable of eating relatively large food items, but many other items are also found in their diet [5]. The large size of ceratophryid tadpoles compared to most sympatric tadpoles could contribute to their propensity toward anurophagy during larval and froglet stages in *Chacophrys*.

Some neuroendocrine regulators (neuropeptid Y, corticotropine-realising factor, α melanocyte-stimulating hormone, corticosterone, and leptine) are associated with food intake, behaviors, and the cessation of feeding in different periods of larval development [23, 24]. Like most anurans, the cessation of feeding for *Chacophrys* and *Ceratophrys* tadpoles is a distinct metamorphic event evidenced by the empty gastrointestinal tract. Furthermore, the loss of their keratinized mouthparts at metamorphosis precludes ingestion of most items their larvae prey upon. There is one report though of *Ceratophrys ornata* continuing feeding during metamorphic climax based on observations of tadpoles maintained in aquarium and fed *Tubifex* worms, which may not require keratinized mouthparts for capture [25].

*Lepidobatrachus* tadpoles, however, are different. Our data on gut contents suggest they can capture prey during all larval stages, including those at metamorphic climax when the tail shortens and the mouth is remodelling. Indeed prey items found in pre and postmetamorphic *Lepidobatrachus* spp. are similar [5].

To summarize, all ceratophryid tadpoles can consume animal food. Tadpoles of *Lepidobatrachus* eat both large or small prey and what they ingest pre and post metamorphosis are similar. At the end of the larval period, food intake seem to continue without the usual interruption at metamorphosis.

### Visceral gross morphology

In anurans, the extensive larval intestine is eventually remodeled during metamorphosis to become the shorter adult intestinal tract [26, 27]. The patterns observed in *Chacophrys* tadpoles is similar to those reported for most omnivore tadpoles. At the metamorphosis, the gastrointestinal tract is empty, the stomach differentiates and the intestine shortens abruptly.

Distinctive “carnivore” morph in *Spea* is characterized by large jaw muscles, notched mouthparts and a short gut [18]. These features are also observed in *Ceratophrys* tadpoles [12]. Furthermore, *Ceratophrys* tadpoles share with *Chacophrys* (and other anurans) the cranial segment of gut (*manicotto glandulare*), a double coiled intestine and the sequence of metamorphic changes in gross morphology (i.e., intestinal tract is empty, the stomach differentiates and the intestine shortens abruptly).

In contrast, *Lepidobatrachus* tadpoles have a true stomach and a short intestine without double coils. Their metamorphosis does not involve emptying, differentiation, or shortening of the alimentary tract. During larval development, the wall of gastrointestinal tract thickens and the internal surface gains glandular complexity. The absence of metamorphosis of the gastrointestinal tract is unique (or at least unknown) in other anurans with free-living tadpoles.

Pharmacolocological experiments that induced phenotypes, which mimicked the interspecific morphological variation, provide some insights into the evolution of carnivory in ceratophryid lineage [15]. The domain of *Pitx2* expression correlates with the position of the gastroduodenal loop, and this loop is positioned more posteriorly in *Lepidobatrachus* (a pattern that can be reproduced in *Xenopus* and *Ceratophrys* using an RA synthesis inhibitor). From these results, one may assume that decreased RA (retinoic acid) signaling occurred only in *Lepidobatrachus* lineage. Also, as *Lepidobatrachus* and *Ceratophrys* share several TH dependent traits, such as rapid development and precocious pepsinogen production, it is reasonable to presume that TH signaling increased in the ancestor to all ceratophryids [15].

Before the thyroid gland becomes functional, THs (from egg yolk), deiodinases, and TR play different role to produce spatiotemporal in the differentiation of tissues with different TH sensitiveness in early embryos [28–30]. Consistent with this perspective, the histomorphology of the thyroid gland during larval development in *Lepidobatrachus* indicates they present low secretion of endogenous TH [31]. This TH could be complemented by TH precursors or TH derived from eggs and/or larval food producing different tissue responses, such as the early and gradual differentiation of the adult-like gastrointestinal tract***.***

Arguments based in a shift in TH signals at early developmental stages of *Lepidobatrachus* may explain the derived structural features of the gastrointestinal tract.

### Microscopic morphology of the anterior segment of the gastrointestinal tract

*Ceratophrys cranwelli* and *Chacophrys pierottii* larvae show a fundic region, also known as “*glandularae manicotto*” that is typical of anuran larvae [32]. This region has tubular glands of uniform characteristics, mainly of oxyntic cells, different from the oxynticopeptic cells present in postmetamorphic specimens [33]. The anatomical transformation of the gastric area during metamorphosis is extensive and includes thickening of the submucosa and external musculature, deepening of the gastric folds, and an increase in the number and types of gastric glands.

In contrast, the stomach of *Lepidobatrachus* larvae at earlier stages has distinctive gastric glands, large gastric folds, and a well differentiated elastic stomach (with thicker submucosa layer and external musculature). Tubular gastric glands show mucous neck cells that secrete neutral mucopolysaccharides and homogeneous like-oxyntic cells. The presence of mucous neck cells in early tadpoles of *Lepidobatrachus* is a unique feature for anuran larvae. In *C. cranwelli*, *Ch. pierottii* (this study), *C. ornata* these cells differentiate at the end of metamorphosis [25].

During early *Lepidobatrachus* larval development, oxynticopeptic cells with cytoplasmic granules like those present in adult stages appear. However, there is no evidence of zymogen granules in early tadpoles, which could related to glandular maturity. Oxynticopeptic cells capable of synthesizing and secreting pepsins are already present in earlier larvae stages of *Lepidobatrachus* [14].

Tubular gastric glands present uniform features throughout of the larval intestine. These gastric glands are formed by oxyntic cells, different from the oxynticopeptic cells, which are capable of secreting HCl and pepsinogen in adults [33, 34]. Absence of secretion of HCl denotes anuran larvae lack of a true stomach [35].

Further, differences between oxyntic cells and oxynticopeptic cells were also identified with electron microcopy in *Rana temporaria* [34]. Oxyntic cells showed small and dense cytoplasmic granules that could secrete HCl. The endocrine function of cells in the gastric region would seem to develop already in *R. temporaria* tadpoles acquiring a more active function via increment of the number of endocrine cells and the size of secretory granules in carnivorous adults [34–36]. In contrast, other authors generalized the absence of secretion of pepsinogen and/or HCl in anuran larvae [33, 35, 37].

In all postmetamorphic specimens studied so far there is certain regionalization in the tubular gastric glands, and it is common to find mucous cells in the duct, and the oxynticopeptic cells in the alveolar portion. Oxynticopeptic cells can be identified by the presence of acidophilic zymogen granules [38–41] as is the case for *Chacophrys pierottii* (froglets) and *Lepidobatrachus* spp. (larval and postmetamorphic stages). However, oxynticopeptic cells in *C. cranwelli,* which appear at the end of metamorphosis showed acidophilic characteristics, but few zymogenic granules. Zymogen granules were not observed in adult of *Rana aurora* [42].

In *Lepidobatrachu*s tadpoles, the gastric features of the stomach, characteristic of postmetamophic anurans, are well-defined at early stages. In contrast, the glandular differentiation of stomach surface occurs at the end of metamorphosis or later in *Chacophrys* and *Ceratophrys*.

## Conclusions

**Fig. 7:**
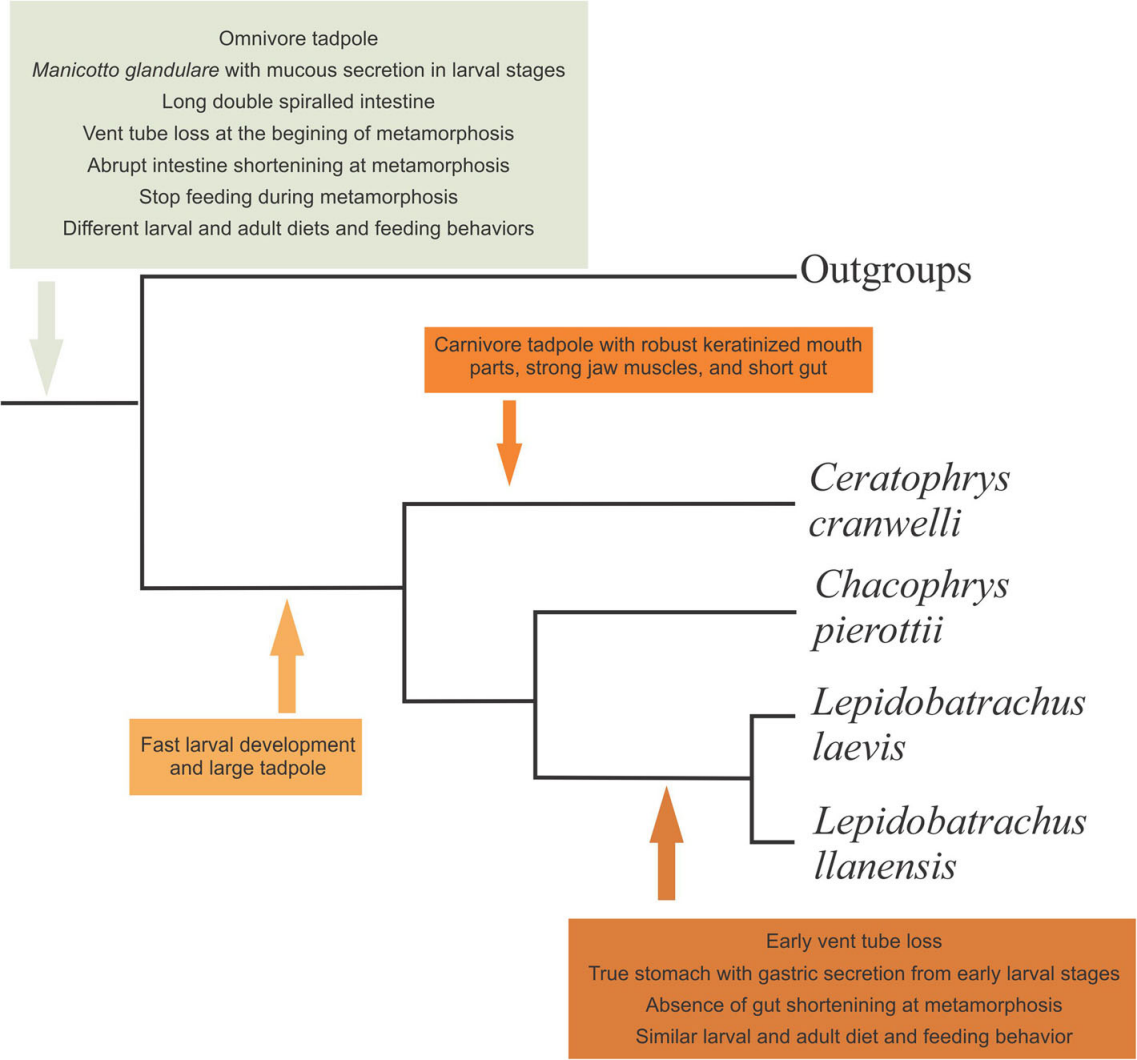
An evolutionary hypothesis regarding the sequence in which developmental variation evolved in ceratophryid lineage with consequences in the suppression of metamorphosis of the gastrointestinal tract in *Lepidobatrachus* studied herein. The phylogenetic relationships were based on molecular data for the whole clade [43]. In comparisons with other frogs, development in extant ceratophryids have accelerated rates of differentiation and growth as larvae which may be influenced by TH signals [13, 15]. Data from the thyroid gland’s histomorphology during larval development indicate however that ceratophryids have low secretory activity at early larval stages suggesting that maternal nutrients and/or diet are possible sources for TH precursors for precocious development [31]. Developmental plasticity led to three larval eco-morphotypes that may breeding simultaneously in the same pond. There is a distinct metamorphosis from the larval to adult gastrointestinal tract in the carnivore tadpole of *Ceratophrys* and the omnivore tadpole of *Chacophrys* similar to what occurs in most anurans*.* In *Lepidobatrachus*, in contrast, the larval gastrointestinal tract is quite similar to that of the adult without abrupt stomach differentiation and intestine shortening at metamorphosis. Furthermore, diet and feeding behavior in larval and adult stages are similar

Figure 7 summarizes the major points of this research. The monophyly of the ceratophryids is well supported by both morphological and molecular data [43]. Rapid larval development, with accelerated growth seems to related with a distinctive TH physiology in the lineage [15, 31, 44].

The extreme trophic morphologies among ceratophryids could had been influenced by many ecological cues (food availability, food quality, pond desiccation, competition). Carnivory in ceratophryid lineage had two major consequences: the carnivore tadpole of *Ceratophrys* and the adult-like carnivore tadpole of *Lepidobatrachus* in which the gastrointestinal metamorphosis disappeared and the only conserved signal of that metamorphosis is the larval vent tube.

It remains to be investigated how much diet and/or exogenous THs have disrupted the ceratophryid development to a direct development of the gastrointestinal tract in the life cycle of *Lepidobatrachus*.

## Methods

The study did not involve experiments, but is a comparative approach, to the anuran metamorphosis in non-model species. The data proceeded from wild caught specimens deposited in herpetological collections and field observations. The study did not included living specimens. Histological sections, anatomical parts from dissections, and gut contents are preserved with similar catalogue numbers to those in the collection.

### Animals

We studied larval and postmetamorphic specimens of *Chacophrys pierottii* (Vellard, 1948)*, Ceratophrys cranwelli* Barrio, 1980*, Lepidobatrachus laevis* Budgett, 1899 and *Lepidobatrachus llanensis* Reig and Cei, 1963. All specimens were collected in ephemeral ponds in various locations in North Argentina in public lands along roads. Specimens were euthanized with overdose of aqueous solution of Tricaine methanesulfonate (MS222, 6 g/l) and fixed in 10% formalin or Bouin’s fixative in the field.

All procedures to collect and kill specimens in the field comply with the legal requirements of current laws in Argentina (approved research protocol PICT 510). Secretaría de Medio Ambiente y Desarrollo Sustentable, Gobierno de la Provincia de Salta, República Argentina, provided authorization to collect field specimens (collection licences N°199,886/2011 and 227–216,600/064/2016). All specimens were accessioned as lots and are deposited in the Herpetological Collection of IBIGEO (IBIGEO-A) as is listed:

- 32°15′08.33″S, 63°07′00.09″W: *Chacophrys pierottii:* IBIGEO-A 1029, 64 larval specimens (Gosner Stages 37–46) collected 28/01/2006; IBIGEO-A 987, 7 adult specimens collected 19/12/2004; IBIGEO-A 2231, 96 larval specimens (Gosner Stages 26–32). *Lepidobatrachus llanensis*: IBIGEO-A 1122, 13 larval specimens (limb growth and metamorphic stages) collected 28/01/2006; IBIGEO-A 2277, 17 larval specimens (digit differentiation stages) collected 20/01/2018; IBIGEO-A 2279, 19 larval specimens (limb growth stages) collected 26/01/2018
- 28°43′59.85″S, 63°25′56.53″W: *Chacophrys pierottii:* IBIGEO-A 2288, 48 larval specimens (Gosner Stages 37–46) and IBIGEO-A 2289 postmetamorphic individuals collected 30/01/2015
- 23°14′49.78″S, 63°21′18.11″W: *Lepidobatrachus laevis:* IBIGEO-A 1322, 57 larval specimens (limb growth stages) collected 08/12/2009. *Lepidobatrachus llanensis:* IBIGEO-A 1321, 23 larval specimens (limb growth stages) collected 08/12/2009.
- 23°16′19.41″S, 63°17′12.20″W: *Lepidobatrachus laevis:* IBIGEO-A 2286, 18 larval specimens (digit differentiation and limb growth stages) and 3 postmetamorphic specimens collected 09/02/2019; IBIGEO-A 2287, 22 larval specimens (limb growth and metamorphic stages) collected 17/02/2019. *Ceratophrys cranwelli*: IBIGEO-A 2282, 22 larval specimens (Gosner Stages 33–39) collected 17/02/2019.
- 23°13′28.23″S, 63°49′26.63″W: *Lepidobatrachus laevis:* IBIGEO-A 1613, 13 larval specimens (digit differentiation stages) collected 12/01/2016. *Lepidobatrachus llanensis:* IBIGEO-A 2275; 78 larval specimens (limb bud stages) collected 13/01/2010.
- 23°12′44.36″S, 63°34′00.13″W: *Lepidobatrachus laevis:* IBIGEO-A 2291, 23 larval specimens (digit differentiation and limb growth stages), IBIGEO-A 2274, 165 larval specimens (limb bud stages) collected 01/11/2000. *Lepidobatrachus llanensis:* IBIGEO-A 567, 19 larval specimens (digit differentiation and limb growth stages) collected 01/12/1998. *Ceratophrys cranwelli:* IBIGEO-A 2290, 13 larval specimens (digit differentiation and limb growth stages) collected 27/12/2007.
- 23°21′50.31″S, 63°6′57.32″W: *Lepidobatrachus laevis:* IBIGEO-A 2276, 15 larval specimens (digit differentiation and limb growth stages) collected 09/02/2019. *Lepidobatrachus llanensis:* IBIGEO-A 2281, 7 larval specimens (digit differentiation and limb growth stages) and 4 postmetamoprhic specimens collected 09/02/2019.
- 24°43′57.59″S, 64°11′49.46″W: *Ceratophrys cranwelli:* IBIGEO-A2283, 78 larval specimens (Gosner Stages 35–41) collected 11/11/1997
- 24°55′58.66″S, 63°39′51.50″W: *Ceratophrys cranwelli:* IBIGEO-A 1937, 75 larval specimens (Gosner Stages 37–43) collected 16/02/2018.
- 22°31′11.65″S, 63°45′59.55″W: *Ceratophrys cranwelli:* IBIGEO-A 21, 45 larval specimens (Gosner Stages 26–33) collected 05/12/1991.

### Analysis

Larval development was staged according to the standard table for anuran embryos and larvae [17] and studied specimens were at larval stages corresponding to the stages: limb bud [26–30], digit differentiation [31–36], limb growth [37–41] and tail shortening (42–46).

Data were obtained from several sources: 1) variation of external morphology during the ontogeny. The ontogeny was described by observing the complete series of development using a stereomicroscope. To document growth, measurements of snout-vent length and total length (snout-tail tip length) in larvae were made with dial calipers (0.02 mm). These measurements are given in millimeters; 2) dissection of larval specimens of *Ceratophrys cranwelli* (*n* = 10), *Chacophrys pierottii* (*n* = 12), *Lepidobatrachus laevis* (*n* = 48), and *Lepidobatrachus llanensis* (*n* = 29) to analyze gut contents. We counted and/or identified items in the stomachs or cranial segment of the intestine to the lowest taxonomic level possible, 3) descriptions of gross visceral morphology and changes in the gastrointestinal tract in dissected specimens, and 4) histological sections of selected specimens. Specimens at stages of digit differentiation, limb growth, advanced metamorphosis, or recently metamorphosed were selected. Descriptions, illustrations, and photographs were made with Nikon SMZ1000 stereo dissection microscope and a Nikon E200 microscope. Photomicrographs were obtained with digital camera adapted.

### Histology protocols

The cranial segment of the gut or stomachs were removed and processed according to the standard histological techniques. Stomach fragments were dehydrated in ascending ethanol series (50–100%), cleared in xylene, and embedded in paraffin. The tissue blocks were serially cross-sectioned at 5 μm with a semiautomatic microtome (Leica RM 2245). Serial slices were stained with the Masson’s trichrome method [45]. Combined Alcian Blue (AB) and periodic acid Schiff (PAS) methods were employed for the demonstration of acidic glycoconjugates and neutral glycoconjugates, respectively [45].

## Data Availability

The datasets used and/or analysed during the current study are available from the corresponding author on reasonable request.
